# Double-lumen tube intubation using video laryngoscopy causes a milder cardiovascular response compared to classic direct laryngoscopy

**DOI:** 10.12669/pjms.321.9044

**Published:** 2016

**Authors:** Wei Wei, Ming Tian

**Affiliations:** 1 Wei Wei, Department of Anesthesiology, Beijing Friendship Hospital, Capital Medical University, Beijing 100050, China; 2 Ming Tian, Department of Anesthesiology, Beijing Friendship Hospital, Capital Medical University, Beijing 100050, China

**Keywords:** Video laryngoscope, Double-lumen tube, Intubation, Hemodynamic response

## Abstract

**Objective:**

To determine whether there is a clinically relevant difference between the circulatory responses to double-lumen tube intubation (DLTI) with the GlideScope video laryngoscope versus the Macintosh direct laryngoscope.

**Methods:**

Eighty adult patients requiring double-lumen tubes for thoracic surgery were randomly and equally allocated to either a Macintosh direct laryngoscope group (DL group, *n* = 40) or a Glide Scope video laryngoscope group (GS group, *n* = 40). DLTI was performed after airway evaluations and induction of anesthesia. Systolic blood pressure (SBP) and heart rate (HR) were recorded before induction (baseline values), immediately before intubation (post-induction values), at intubation and after intubation. Rate-pressure-product (RPP), and the areas under SBP- and HR-time curves were calculated. All data obtained by the two devices were compared.

**Results:**

After laryngoscope insertion, SBP of DL and GS groups changed significantly differently (13.1% vs. 4.6%, *P*< 0.001), while HR changed similarly (17.2% vs. 14.6%, *P* = 0.074). One minute after intubation, both SBP and HR significantly increased in both groups (SBP: 11.6% vs. 11.9%; HR: 18.4% vs. 10.8%), but there were no significant differences between the two groups. RPP significantly increased in both groups after laryngoscope insertion (32.6%, *P*=0.001; 18.2%, *P*=0.002), and there was a significant difference between the two groups (*P* =0.001). Throughout intubation, the areas under SBP-time curves had a significant difference between the two groups (P = 0.042), while those under HR-time curves did not differ significantly (P=0.06).

**Conclusion:**

The intubation response was most significant upon laryngoscope insertion during the whole intubation process. The GlideScope video laryngoscope induced milder circulatory fluctuations than the Macintosh direct laryngoscope did, suggesting that DLTI using video laryngoscopy can help reduce the cardiovascular response to intubation.

## INTRODUCTION

Anesthetists are alwayschallengedfor determining whendifficult intubationoccurs,^1^ which may be solved by video laryngoscopy. Of all currently available video laryngoscopes, the GlideScope video laryngoscope(Verathon Inc., Bothel, WA, USA) is most representative. With a unique design that shows the laryngeal structure on a display screen though optical fibers, it can clearly expose the larynx and ease the operation of tracheal intubation.^2^,^3^ Currently, most studies involving the GlideScope video laryngoscope have focused on simplification of intubation but the effects on hemodynamic fluctuations or the injuries to teeth and laryngeal tissues have seldom been studied.^4^,^5^

Endotracheal intubation, which strongly stimulates various oral organs and tissues, causes hemodynamic fluctuation that cannot always be eliminated by anesthesia induction. The response to endotracheal intubation results primarily from direct stimulation of laryngoscope.^6^ In addition, hemodynamic changes during intubation may alter depending on the operator’s skill in manipulating the laryngoscope.^7^ Therefore, improving airway tools or some other technologies can relieve intubation response by reducing the stimulation.^8^,^9^ Since in thoracic surgery, the double-lumen endotracheal tube required for lung isolation has large outside diameter, low flexibility and high rigidity, double-lumen tube intubation (DLTI) is much more difficult than single-lumen tube intubation, even in patients with normal airways.^10^,^11^ During DLTI, anesthetists often need to exert a greater force to employ combined techniques, which dramatically affects the circulation.^12^,^13^

In order to alleviate the response to DLTI, the GlideScope video laryngoscope has been widely used in clinical settings. Possessing a wide-angle lens and external display function, this laryngoscope can improve the visual field, increase the success rate of intubation,^14^,^15^ shorten the DLTI time and reduce damages.^16^,^17^ However, whether DLTI using the GlideScope video laryngoscope can reduce the circulatory fluctuations caused by laryngoscope stimulation compared with that using the direct laryngoscope remains unknown, although Russell et al. reported that the former laryngoscope produced a lower pressure on tissues inside the mouth.^18^ The aim of this study was to compare the hemodynamic responses to DLTI using direct laryngoscope and video laryngoscope.

## METHODS

### Patients

Eighty patients (aged 18-65 years old, ASA grades I-III) who underwent elective thoracic surgery were included in this study. It was approved by the ethics committee of our hospital, and written consent has been obtained from all patients. Exclusion criteria were obesity (BMI>30), history of difficult intubation, mouth opening less than 3 cm, and failure in the first intubation. Patients were routinely fasted for eight hours before surgery. After the patients entered the operating room, airway evaluations were performed for BMI, Mallampati classification, mouth opening, and neck mobility. Afterwards, the patients were randomly divided into a Macintosh direct laryngoscope group (DL group, *n* = 40) and a GlideScope video laryngoscope (Verathon Inc., Bothel, WA, USA) group (GS group, *n* = 40). The two groups were comparable with regard to demographic data and airway evaluations (Table-I).

**Table-I:** Patient demographic data.

	GS group	DL group
Age; years	57.2 (5.4)	60.1 (8.7)
Male / female	21 / 19	17 / 23
Weight; kg	62.4 (12.0)	60.1 (9.5)
Height; cm	165.6 (8.4)	168.0 (6.8)
BMI; kg/m^2^	21.0 (5.6)	21.3 (3.4)
ASA; 1 / 2	12 / 18	14 / 16
Mallampati	I-II	I-II

### Methodology

Datex-Ohmeda S/5 anesthesia monitor (DatexInstrumentarium, Helsinki, Finland) was used to monitor electrocardiogram and pulse oxygen saturation. Radial artery cannulation was performed to monitor continuous arterial pressure. Double-lumen endotracheal tubes (Hi-contou, Mallincrodt Medical, Athlone, Ireland) were initially lubricated with lidocaine cream (Batch No. 070105; Beijing Zizhu Pharmaceutical Co., Ltd., Beijing, China), and the proximal tip was curved by approximately 90°. After pre-oxygenation, 2 mg midazolam, 0.5μg·kg^-1^ sufentanil, 1.5 mg·kg^-1^ propofol and 0.8 mg·kg^-1^ rocuronium were intravenously administered. Ninety seconds after rocuronium administration, the direct laryngoscope or GlideScope video laryngoscope was inserted for intubation. Left double-lumen tubes (F35) were used. Systolic blood pressure (SBP) and heart rate (HR) were recorded after the patients entered the operating room (T1), after anesthesia induction (T2), when the glottis was exposed (T3), when the tube went into the glottis (T4), and one minute after intubation (T5).

### Statistical analysis

Rate-pressure-product (RPP) and the areas under the SBP- or HR-time curve (AUC) were calculated. SPSS Statistics 19.0 software (International Business Machines Corporation, Armonk, NY, USA) was used for statistical analyses. Numerical data comparisons were performed using repeated measures analysis of variance or independent-samples *t*-test. Categorical data were compared using the Chi-square test.

## RESULTS

### Demographic data and airway evaluations

There were no significant differences in the demographic data or airway evaluations.(Table-I)

### Impact of laryngoscope and intubation on SBP and HR

**Fig.1:**
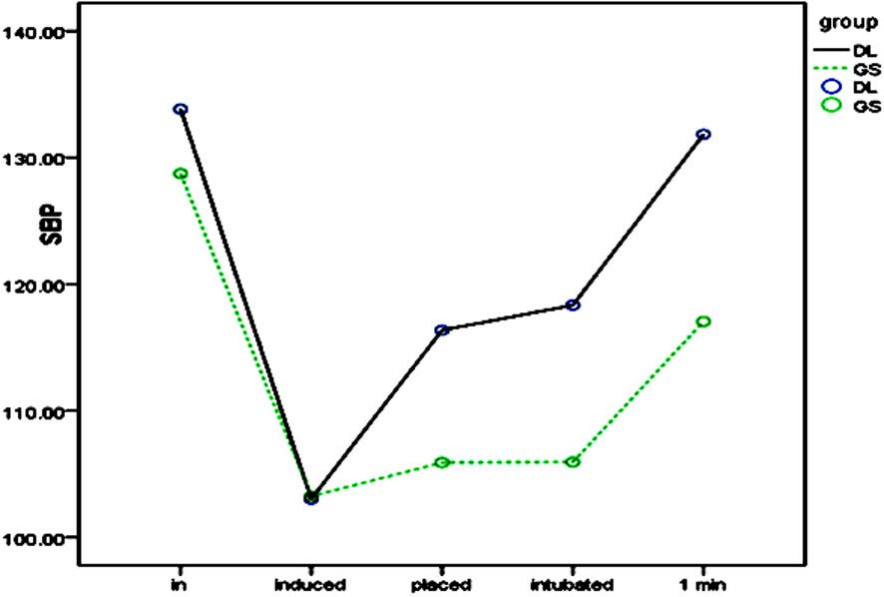
SBP at different time points.

SBP and HR after entering the operating room (T1) showed no significant differences between the two groups. Fig-1 and Fig-II. After anesthesia induction (T2), SBP and HR decreased significantly in both groups, with significant inter-group differences. Upon glottis exposure (T3), SBP significantly increased in the DL group but showed no significant difference in the GS group. HR significantly increased in both groups, with a significant inter-group difference. SBP and HR at intubation (T4) did not change significantly compared with those at T3. However, one minute after intubation (T5), SBP significantly increased in both groups, especially in the DL group, butHR increased similarlyin the two groups.

**Fig.2:**
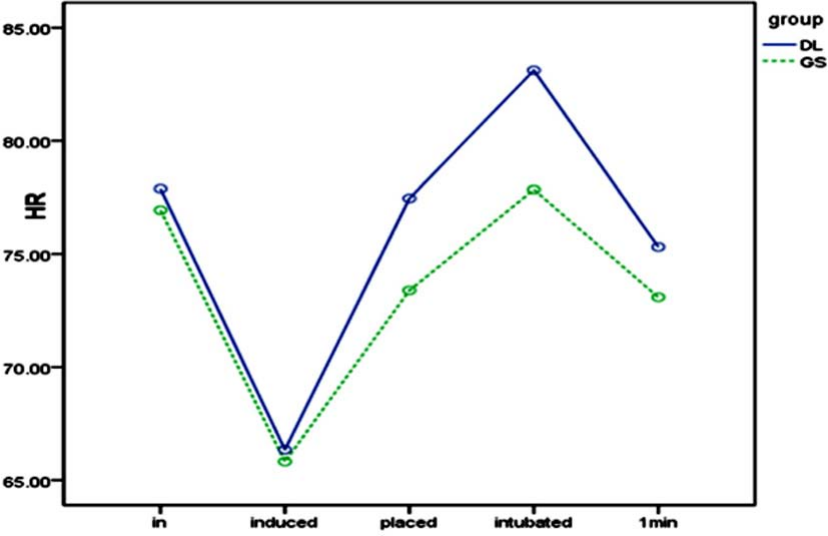
HR at different time points.

### Changes in RPP at laryngoscope insertion and intubation

**Fig.3:**
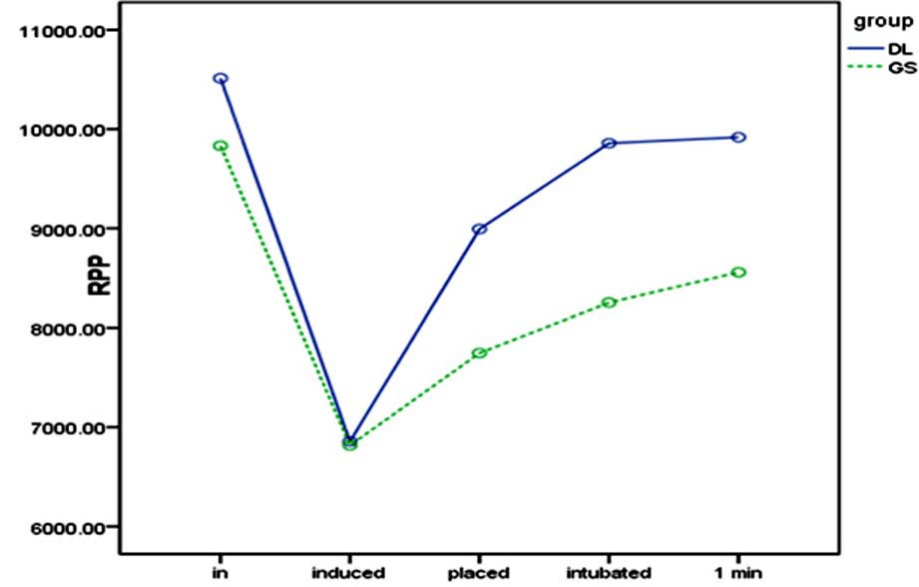
RPP at different time points.

After anesthesia induction (T2), RPP dropped significantly in both groups, with a significant inter-group difference though (Fig.3). Upon glottis exposure (T3), RPP significantly increased in both groups, particularly in the DL group. At intubation (T4), RPP significantly increased in the DL group but showed no significant difference in the GS group. One minute after intubation (T5), RPP showed no significant changes in either group.

### Changes inSBP and HR over time

The area under the SBP-time curve (AUC_SBP_) and the area under the curve when SBP reached maximum (AUC_MAXSBP_) were significantly different between the two groups (*P*=0.042, 0.001). Table-II. In contrast, the area under the HR-time curve (AUC_HR_) and thearea under the curve when HR reached maximum (AUC_MAXHR_) were similar in the two groups (*P*=0.06, 0.138).

**Table-II:** Comparisons of AUC values between the two groups.

		t	df	Sig.(2-tailed)
SBP	AUC	2.077	59.979	0.042
SBP	AUCmax	6.239	44.6	0
SBP	AUCrecovery	4.717	58.493	0
HR	AUC	1.91	68	0.06
HR	AUCmax	-1.502	68	0.138
HR	AUCrecovery	2.028	54.03	0.047

## DISCUSSION

Decrease of cardiovascular responses during intubation has been spotlighted in clinical anesthesia. The GlideScope video laryngoscope enjoys clear display of the larynx or the glottis, facile operation and easy intubation.^19^,^20^ Cardiovascular responses become more intense with prolonged intubation, which may endanger the patients with hypertension.^5^

During endotracheal intubation, laryngoscope stimulates sympathetic nerves in the oral cavity and the trachea. As a result, considerable catecholamine is released, and SBP and HR are elevated in patients.^21^,^22^ In this study, BP and HR at endotracheal intubation significantly increased compared with those after anesthesia induction (T2), suggesting that anesthetic drugs failed to eliminate the stimulations caused by the laryngoscope and endotracheal tube. For the elderly or critically ill patients, other auxiliary methods, such as local anesthetic spraying and application of beta-receptor antagonists or calcium channel blockers, should be used to reduce intubation stimuli.^12^,^23^

During the whole process of intubation, stimuli result mainly from insertion of laryngoscope to expose the glottis and passing of endotracheal tube through the glottis. Similar to the results of Hassan et al.^22^ we found that circulatory changes occurred mainly when the laryngoscope was inserted. At this time, SBP and HR in GS and DL groups increased. In contrast, SBP and HR barely changed in the two groups when the endotracheal tube was inserted, so laryngoscope stimulation was mainly responsible for the circulatory fluctuations during intubation, being consistent with the conclusion of Yangetal.^24^ Thus, changing intubation tools may relieve the response to intubation. Compared with the DL group, SBP and HR showed milder changes after the insertion of laryngoscope in the GS group, suggesting that video laryngoscope relieved the stimulation of oral tissues and mitigated the intubation response. Takahashi et al. have reported similar findings.^9^ Lee et al.^7^ pointed out that this may be related to the lower pressure of GlideScope video laryngoscope on tissues in the oral cavity under the same conditions upon glottis exposure. Russell et al. also confirmed this by measuring the compressive force on the back of the tongue.^18^

The optical fiber lens of the GlideScope video laryngoscope make doctors’ view forward, and the wider angle blade reduces the pressure of laryngoscope on the tongue body, pharyngeal wall and epiglottis, thus increasing the success rate of intubation.^25^ This effect is more obvious for double-lumen tubes that have large diameter, high rigidity and lowflexibility.^11^,^16^ However, some researchers claim that wide angle lens renders the entry of the endotracheal tube into the glottis difficult, which often needs assistance, and that intubation using the GlideScope video laryngoscope hardly affects the increase in the myocardial oxygen consumption load (RPP) caused by intubation stress.^26^,^27^ In this study, after the laryngoscope was inserted, increase in RPP of the GS group was less significant than that of the DL group; however, when the double-lumen tube was inserted, RPP increased in the GS group, but did not change significantly in the DL group. Hence, although the GlideScope video laryngoscope relieved laryngoscope stimulation, the stress reaction upon the insertion of double lumen tube into the glottis was stronger. Probably, the angle of laryngoscope blade created different paths for the endotracheal tube to enter the glottis, so the double-lumen tube stimulated the glottis and throat of the GS group more apparently.

After the double-lumen tube entered the glottis, circulatory indices in the two groups did not change obviously, without significant inter-group differences also. However, the area under the SBP-time curve (AUC_SBP_) showed a significant difference between the two groups (*P* = 0042), suggesting that although GlideScope did not significantly reduce catheter stimuli, it managed to mitigate the circulatory response during intubation by decreasing the pressure of laryngoscope, improving the glottis exposure, and reducing the intubation time. Contrarily, since the area under the HR-time curve (AUC_HR_) showed no significant difference between the two groups, laryngoscopic operation and catheter stimuli mainly caused SBP and HR changes respectively. This finding is consistent with the result of Kovac.^28^

In summary, DLTI using video laryngoscopy caused a milder cardiovascular response than that using classic direct laryngoscopy did.
